# New Medical Schools

**Published:** 1924-09

**Authors:** 


### NEW MEDICAL SCHOOLS.

One of the most valuable of recent efforts for the treatment of mental disease has been the establishment of the Maudsley Hospital with its pathological department for the purpose of research in psychological medicine. Started at the suggestion of Dr. Henry Maudsley, who gave altogether £40,000 towards the cost of building, it was erected for the teaching of this subject, and for the treatment, on a voluntary basis, of recoverable cases of mental disorder and the neuroses. There are 157 beds available in wards and separate rooms, together with an out-patients' department. The hospital has now been admitted as a school of the University of London.

The pathological department associated with it serves all the London County Mental Hospitals and includes a library and laboratories for anatomical, biochemical, bacteriological and experimental research into the pathology of nervous and mental disorders. There students may work, usually free of charge, when permission has been obtained from the Director of the Pathology Department, Dr. F. L. Golla, M.B., F.R.C.P. ; permission to work in the Hospital may be obtained from the Medical Superintendent, Mr. Edward Mapother, M.D., M.R.C.P.,, F.R.C.S. There are also courses of instruction for those desiring to take a Diploma in Psychological Medicine. Thus students have every facility for close, wide, fruitful research. In addition to the Maudsley Hospital Bethlem Royal Hospital has now been constituted a School of the University for similar purposes. The importance of these additions to teaching power on this important subjec need not be insisted upon.

